# Hernie post traumatique de la paroi abdominale antérieure

**DOI:** 10.11604/pamj.2016.24.203.9517

**Published:** 2016-07-08

**Authors:** Zeineb Mzoughi, Rached Bayar, Hamdi Khmiri, Lassad Gharbi, Mohamed Taher Khalfallah

**Affiliations:** 1 Université de Tunis El Manar, Faculté de Médecine de Tunis, 1007, Tunis, Tunisie, Service de Chirurgie Digestive et Hépatobiliaire, CHU Mongi Slim, La Marsa

**Keywords:** Hernie, traumatisme, laparoscopie, prothèse, Hernia, trauma, laparoscopy, prosthesis

## Abstract

La hernie post-traumatique de la paroi abdominale antérieure peut être méconnue dans un contexte d’urgence. Nous rapportons l’observation d’un patient âgé de 32 ans, avec un BMI à 30 Kg/m^2^ ayant présenté une hernie de la paroi abdominale antérieure suite à un accident de la voie publique. Cette lésion était méconnue par l’examen clinique. La tomodensitométrie abdominale montrait un défect de 8 cm de la paroi abdominale antérieure. Le patient était opéré avec découverte d’un défect musculo-aponévrotique sur 12 cm. La réparation était réalisée par une suture par des points séparés. Les suites opératoires étaient marquées par une nécrose secondairement infectée de la peau. Elle avait bien évolué après cicatrisation dirigée. A 3 mois post-opératoire, le patient va bien avec une plaie cicatrisée et une paroi abdominale solide.

## Abstract (en)

Post traumatic anterior abdominal wall hernia can be ignored in emergency settings. We here report the case of a 32-year-old patient with a BMI of 30 kg/m^2^, suffering from anterior abdominal wall hernia as a result of a road accident. This lesion wasn’t detected during clinical examination. Abdominal tomodensitometry showed a defect of 8 cm in the anterior abdominal wall. The patient underwent surgery during which a musculoaponeurotic defect of 12 cm was detected. The repair was carried out using interrupted suture. The postoperative course was marked by a secondarily infected skin necrosis. The evolution was satisfactory after directed cicatrization. At 3 months postoperatively the patient was doing well with a healed wound and a strong abdominal wall.

## Introduction

La hernie post traumatique de la paroi abdominale est une entité rare. Elle est souvent méconnue dans un contexte d’urgence.

## Patient et observation

**Figure 1:**
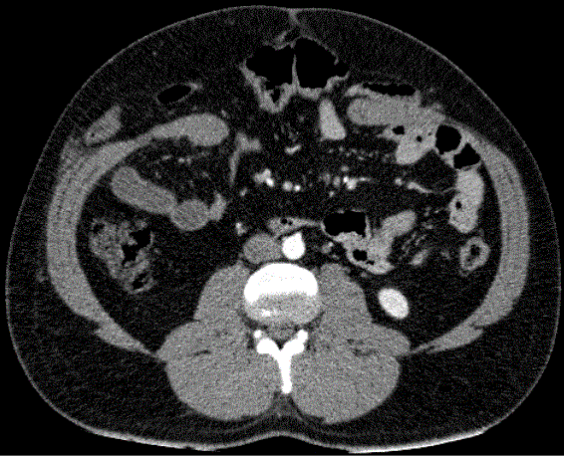
Tomodensitométrie abdominale montrant un defect de la paroi abdominale antérieure avec des anses digestives sous la peau

**Figure 2:**
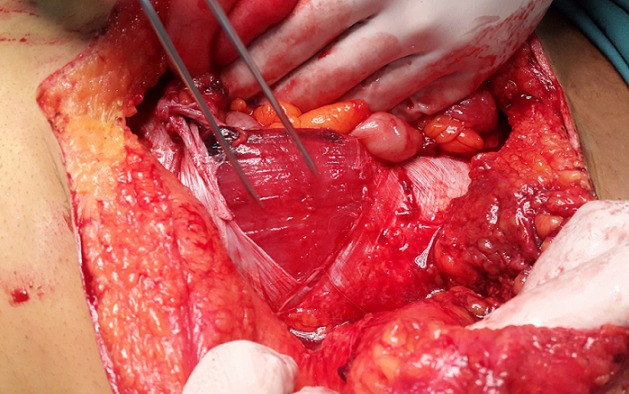
Vue per opératoire montrant la rupture de l’aponévrose antérieure des grands droits et l’issue de l’intestin grêle

Un patient âgé de 32 ans, sans antécédents pathologiques, a consulté aux urgences suite à un accident de la voie publique. Il était percuté par un camion avec écrasement abdominal contre le volant. A l’examen, il était stable sur le plan hémodynamique et respiratoire. Il avait un BMI à 30 Kg/m^2^. L’examen abdominal trouvait des lésions de dermabrasion en péri ombilical. L’abdomen était distendu, sensible dans son ensemble. La biologie était normale. La tomodensitométrie(TDM) thoraco-abdominale montrait un défect de 8 cm de la paroi abdominale antérieure avec une rupture de l’aponévrose des grands droits et des anses digestives sous la peau (Figure 1). Il n’y’avait pas d’épanchement intraabdominal liquidien ou gazeux. On avait décidé d’opérer le patient par voie médiane. A l’exploration, il existait un décollement du plan sous cutané, avec un défect musculo-aponévrotique de 12 cm. Les anses grêles n’étaient plus recouvertes que par le péritoine pariétal (Figure 2). Il y’avait un hémopéritoine de faible abondance en rapport avec une plaie du méso du grêle à 50 cm de la valvule de bauhin. La fermeture du plan aponévrotique était réalisée par une raphie par des points séparés au vicryl 1 en essayant de rapprocher les berges de l’aponévrose des grands droits. Les suites opératoires étaient marquées par la survenue à J3 post opératoire d’un abcès de la paroi ayant bien évolué après une cicatrisation dirigée. La sortie était autorisée au bout de 13 jours postopératoires. Actuellement, le patient est à 3 mois de l’intervention, il va bien avec une plaie cicatrisée et une paroi abdominale solide.

## Discussion

La hernie post traumatique(HPT) de la paroi abdominale se divise en 3 types: type I petit defect, type II grand defect, type III grand defect avec issue d’organes intraabdominaux comme dans notre cas [1]. Le type III est rare et souvent dû à un mécanisme de décélération. Cette lésion pariétale post-traumatique peut être méconnue surtout lorsqu’il s’agit d’un type I et tout particulièrement dans un contexte d’urgence. Chez notre patient obèse, la hernie n’était pas visible à l’examen qui était focalisé à la recherche d’une lésion intraabdominale traumatique. L’intérêt de la TDM est indéniable. Elle permet de mettre en évidence la hernie et de rechercher des lésions post traumatiques associées. Le traitement peut être différé si la lésion est non détectée ou non compliquée. Il consiste en une réparation prothétique par voie laparoscopique dans la majorité des cas. Si la lésion nécessite un traitement urgent du fait de lésions associées (30%) ou en cas d’étranglement rare, la réparation se fait par une autoplastie ou une plaque biface [2].

## Conclusion

Une HPT abdominale antérieure doit être suspectée surtout devant un mécanisme de décélération. La clinique pouvant faire défaut, le scanner abdominal pose le diagnostic. La prise en charge chirurgicale immédiate ou différée dépend des lésions abdominales associées.
